# Bioluminescence Tomography Based on One-Dimensional Convolutional Neural Networks

**DOI:** 10.3389/fonc.2021.760689

**Published:** 2021-10-18

**Authors:** Jingjing Yu, Chenyang Dai, Xuelei He, Hongbo Guo, Siyu Sun, Ying Liu

**Affiliations:** ^1^ School of Physics and Information Technology, Shaanxi Normal University, Xi’an, China; ^2^ School of Information Sciences and Technology, Northwest University, Xi’an, China

**Keywords:** bioluminescent tomography (BLT), optical reconstruction, deep learning, convolutional neural networks, inverse problem

## Abstract

Bioluminescent tomography (BLT) has increasingly important applications in preclinical studies. However, the simplified photon propagation model and the inherent ill-posedness of the inverse problem limit the quality of BLT reconstruction. In order to improve the reconstruction accuracy of positioning and reconstruction efficiency, this paper presents a deep-learning optical reconstruction method based on one-dimensional convolutional neural networks (1DCNN). The nonlinear mapping relationship between the surface photon flux density and the distribution of the internal bioluminescence sources is directly established, which fundamentally avoids solving the ill-posed inverse problem iteratively. Compared with the previous reconstruction method based on multilayer perceptron, the training parameters in the 1DCNN are greatly reduced and the learning efficiency of the model is improved. Simulations verify the superiority and stability of the 1DCNN method, and the *in vivo experimental* results further show the potential of the proposed method in practical applications.

## 1 Introduction

Bioluminescence tomography (BLT) is an optical molecular imaging method with high sensitivity, low cost, and noninvasive characteristics (1–3). Traditionally, based on the light propagation model in biological tissues, the inversion algorithm is used to recover the three-dimensional (3D) distribution of the internal bioluminescent sources that enables quantitatively monitoring the pathological and physiological changes of the biological entities (4). In the past decade, BLT has been widely applied in preclinical studies such as early detection of tumors, monitoring tumor growth, and metastatic spreading (5–8).

For most BLT applications, both tumor spatial location and morphology are the key problems need to be addressed. However, the light scattering and limitation of measurement strongly influence the reconstruction accuracy. Considering the ill-posedness of BLT reconstruction and the sparseness of the source distribution, researchers have proposed various reconstruction algorithms combined with different prior information (9–13). Although the positioning accuracy of the reconstructed source center is gradually improved by these methods, the insufficient sparseness of the reconstructed results would lead to image artifacts and limit the accuracy of morphological analysis.

Deep-learning methods have become a dominant methodology of choice for analyzing medical images and medical imaging in the past few years (14, 15). They have shown outstanding performance on solving a variety of inverse problems (16). Recently, deep-learning methods have also received increasing attention in optical molecular tomography. Yoo et al. proposed an encoder-decoder convolutional structure deep neural network for diffuse optical tomography (DOT) (17). The experimental results demonstrated that the trained network performed well and could obtain accurate locating results in regular phantom without iterative procedure or linear approximation. Huang et al. proposed a deep convolutional neural network, gated recurrent unit, and multiple-layer perception-based method (18) to improve the quality of fluorescence molecular tomography (FMT) reconstruction. Wang et al. proposed an inverse-problem solving technology based on a stacked autoencoder (SAE) network for FMT (19). Simulation based on a uniform two-dimensional rectangular model shows the proposed method can retrieve the positions and shapes of the targets accurately. Lin et al. proposed a three-dimensional deep encoder-decoder network for FMT (20), which achieved accurate locating results in regular phantom. Gao et al. proposed a multilayer perceptron-based inverse problem simulation (IPS) method, which is the first deep-learning method applied to BLT (21). The simulations and *in vivo* experiments demonstrated that the IPS method has advantages over the traditional direct analysis and the iterative methods. However, due to the complexity of a fully connected layer connection, the network training for IPS needs too many parameters, and it is also difficult to transmit the gradient during training especially when the fully connected network layer is deep.

In this study, a deep-learning method based on one-dimensional convolutional neural networks (1DCNN) is proposed for BLT. It does not rely on an analytic inversion or on an iterative data-fit optimization. Here, we use the term 1DCNN to emphasize that the input of the CNN is a one-dimensional vector of the surface measurement. Unlike the IPS method, the local connection and weight sharing of CNN greatly reduce the number of parameters to be trained in the neural network model. Simulations and *in vivo* experiments with a mouse brain orthotopic glioma model are performed to verify the performance of the proposed method in BLT reconstruction.

This paper is organized as follows. In *Section 2*, the 1DCNN network-based reconstruction method, the design of data collection, and the evaluation index are explained. Simulations are then presented to verify the reconstruction ability of the proposed method in *Section 3*. *Section 4* further evaluates the proposed method with *in vivo* experiments. Finally, we present a discussion and conclusion in *Section 5*.

## 2 Methodology

### 2.1 BLT Reconstruction Based on 1DCNN Method

Here, we present a data-driven reconstruction method based on deep learning. Unlike the model-based method, neural networks (NNs) form the theoretical architecture of deep-learning methods. The universal approximation theorem (22) guarantees that a NN with sufficiently many hidden units and a linear output layer is capable of representing any arbitrary function. The CNN is one of the most representative algorithms of deep learning, which is a kind of feed-forward neural network including convolution calculation and deep structure. Researches have shown that the convolutional layer can extract high-level features from data and obtain more useful information (23). Therefore, it is possible to use the CNN to solve the inverse problem of BLT by directly fitting the nonlinear mapping relationship between the surface photon flux density and the distribution of the internal bioluminescence sources.

**Figure 1:**
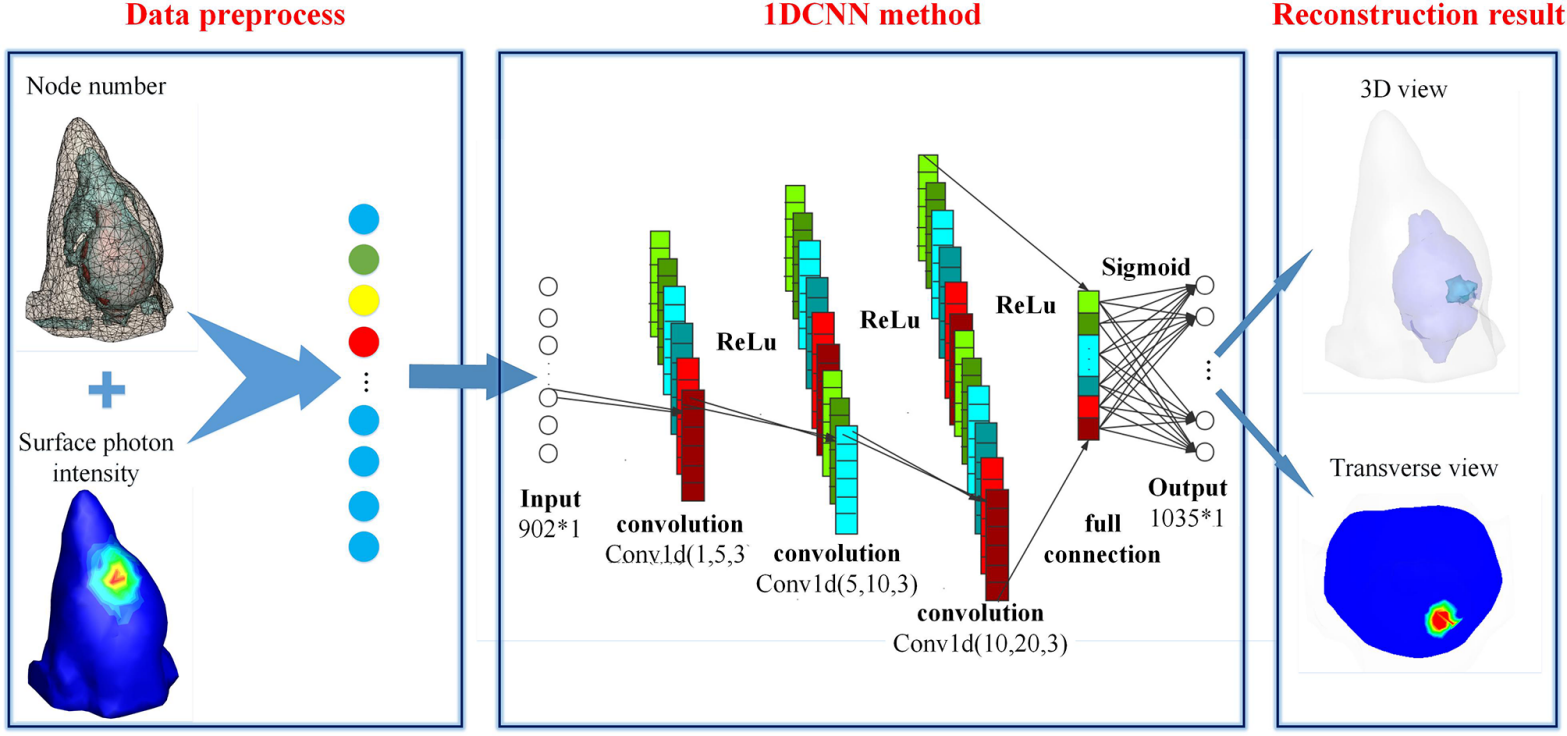
The schematic diagram of 1DCNN used in BLT reconstruction.

**Figure 1** shows the schematic diagram of 1DCNN used in BLT reconstruction. Basically, the 1DCNN is an end-to-end learning model including six layers, i.e., an input layer, three convolutional layers, a fully connected layer, and an output layer. The model task of 1DCNN is to extract the characteristic information of the surface photon flux density and predict the spatial distribution of the internal source, which is different from that of general classification problems. Considering that the commonly used pooling mechanism may change the structure information and thus affect the reconstruction results, we deprecated the pooling mechanism in the 1DCNN. The input to the deep network is an *N*-tuple vector of photon flux density *ϕ*, where *N* is the number of surface nodes. It is a vector with characteristic information, which is obtained by arranging the elements of measurement according to the order of surface nodes in data preprocessing stage. The output of the 1DCNN is an *M*-tuple vector of the reconstructed source *S*, where *M* is the number of nodes in the imaging region. By combining the output with the coordinate information of nodes, the 3D distribution of internal source can be obtained.

As illustrated in **Figure 1**, each convolution layer is followed by an activation function (ReLU). In the leaning system of 1DCNN, the convolution layers are used for feature extraction. The feature maps produced by the previous layer are convolved with several convolutional kernels (to be learned in the training process). The output of the convolution operators along with a bias (to be learned) is passed by the activation function to form the feature map for the next layer. This process can be defined as follows:


(1) $$h_{j}^{l}=g\left(\Sigma_{i=1}^{k}h_{i}^{l-1}*W_{ij}^{l}+b_{j}^{l}\right)$$

where $h_{j}^{l}$ represents the *j*th feature map (*h* ^0^ = *ϕ*) of *l*th convolutional layer, $W_{ij}^{l}$ represents the weight matrix connecting the *i*th feature map of layers *l* – 1 and the *j*th feature map of the layer *l*, *i* and *j* are the indexes of the input and output feature maps, *k* represents the number of feature maps in layers *l* – 1, and $b_{j}^{l}$ is the bias corresponding to each feature map of the layer *l*. *g*(·) is the ReLU activation function:


(2) g(x)=ReLU(x)=max(0,x)

The fully connected layer in **Figure 1** is used to connect all the features and pass the output value to the classifier. The sigmoid function is used as the classifier, and it is defined as follows:


(3) $$\text{Sigmoid}(x)=\frac{1}{1+e^{-x}}$$

During the network training process, the method attempt to fit the nonlinear mapping between the bioluminescence source and the surface photon flux density. The inverse problem of BLT is optimized as follows:


(4) $$\min{\left||f_{1}(\phi |\theta )-S|\right|}_{2}^{2}$$

where *f*_1_ is the 1DCNN method with network weight *θ*. *ϕ* is the surface photon flux density, and *S* is the bioluminescence source. Moreover, the network weight *θ* is updated iteratively during the network training by minimizing the BCE between the actual and reconstructed sources.

The adaptive moment estimation (Adam) optimization function is applied for optimizing the loss function. The training hyperparameters are set as follows: learning rate *α* = 0.001, *β*_1_ = 0.9, *β*_2_ = 0.999, *ϵ* = 10^–8^, epochs = 200, and batch size = 32. The training time of the model is about 5 min. The training parameters of the 1DCNN is about 10^5^, which has been reduced by 83% compared with IPS. The related computing configuration environment of implementing the network model mainly includes Ubuntu 16.04 system, python3.6, and pytorch1.6. The whole calculation procedure ran on a server with Intel(R) Xeon(R) Silver4214CPU @2.20 GHz, 12 GB RAM, and NVIDIA GTX2080 GPU.

### 2.2 Data Collection

There is no doubt that the collection of a large amount of representative data is important for a data-driven reconstruction method. The datasets used in previous studies were mostly obtained based on the Monte Carlo method since data acquisition from *in vivo* experiments is not practical. Although the Monte Carlo method has high reliability due to its statistical characteristics, the cost of time is not insignificant. In our implementation, the simplified spherical harmonics approximation (*SP_N_*) to the radiation transfer equation is solved numerically to generate simulation training datasets. To balance efficiency and accuracy in data collection, we use the finite element method to solve the *SP*_3_ equation (24). It takes about 70 h to obtain the dataset used for the following simulation.

In order to improve the generalization ability of the experiment, the standard digital mouse model (25) was selected. Because the brain glioma is a type of intracranial tumor and only invades inside the brain, we selected the head of the mouse as the reconstruction region, which includes three organs: brain, skull, and muscle. The corresponding optical parameters (26, 27) are presented in **Table 1**. The tetrahedron mesh used for simulations includes 5,831 tetrahedron mesh nodes and 31,826 tetrahedron elements.

**Table 1:** Optical parameters of main organs.

Organ	μ*_a_*/mm^–1^	μ*_s_*/mm^–1^
Brain	0.0389	1.7134
Skull	0.0804	2.0690
Muscle	0.1154	0.4674

Simulation data of single source and dual source were collected to train the 1DCNN and validate the reconstruction performance. Since the internal sources can be anywhere in the mouse brain, the simulated samples should cover brain tissue as much as possible. We randomly selected a node of the brain tissue as the internal bioluminescence source center, and then set the immediate adjacent tetrahedrons containing the center node as a single source. In this way, we obtain the simulated single sources by traversing all nodes in the brain region. Due to the uneven mesh, the simulated single sources were irregular and their shape and size were not exactly the same, but such operation increases the diversity of data samples. The minimum volume of single source is about 3 mm^3^, and the maximum volume of single source is about 25 mm^3^. For the given single source, we can obtain the corresponding surface photon density by solving the *SP*_3_ using FEM. To generate dual-source data, we use a simple combination method, i.e., randomly combining two single-source samples to obtain a dual-source sample. The surface photons density *ϕ* and the internal sources *X* of the assembled source samples were calculated as follows:


(5) $$\phi_{dbs}=\sum_{i\in S_{n}}\phi_{i}$$

(6) $$X_{dbs}\sum_{i\in S_{n}}X_{i}$$

where *S_n_* and *n* are the sets of selected single-source samples and the number of selected samples. *ϕ_i_* and *ϕ_dbs_* are the surface photons of *i*th single- and double-source samples, *X_i_* and *X_dbs_* are the given true bioluminescent sources of the *i*th single- and double-source samples. Dual-source samples were created by randomly selecting two samples (*n* = 2) from the single-source samples. According to the above data collection scheme, a total of 11,635 samples (including 1,035 groups of single-source samples and 10,600 groups of dual-source samples) were generated, in which 1,094 simulation samples were used as the validation sets to determine the optimal model, and 935 simulation samples were used as the test sets to test the model. By introducing double-source samples, the proportion of single-source samples in the training dataset is diluted, and the invisible prior of the model with regard to the number of sources is confused, so as the generalization ability of the model is improved.

### 2.3 Evaluation Index

To justify the utility of the proposed method, simulations and *in vivo* experiments are carried out for BLT reconstruction with 1DCNN in comparison with the IPS method. We use two metrics, the location error (LE) and the Dice index (28), to quantitative evaluate the location accuracy and the morphological similarity, respectively.

The LE is the Euclidean distance between the barycenter of the reconstructed source and that of the true anomaly. The LE is measured as the function:


(7) $$\text{LE}={\left||\text{S}{\text{C}}_{re}-\text{S}{\text{C}}_{tr}|\right|}_{2}$$

(8) $$\text{S}{\text{C}}_{k}=\left(\sum_{i\in S_{k}}P_{i}\times x_{i}\right)/\sum_{i\in S_{k}}x_{i}$$

where SC*_re_* and SC*_tr_* are the barycenter coordinate of the reconstructed source and true source, respectively. ||•||_2_ is the operator of Euclidean distance. SC*_k_* is the weighted center coordinate of source *S_k_*, *P_i_* presents the coordinate vector of the *i*th node in *S_k_*, and *x_i_* is the reconstructed intensity of *P_i_*.

The Dice index reflects the morphological similarity between the nodes set of the reconstructed source and the real light source. The higher the Dice index, the better the morphological similarity.


(9) $$\text{DICE}=\frac{2\left|S_{1}\cap S_{2}\right|}{\left|S_{1}\right|+\left|S_{2}\right|}$$

where *S*_1_ and *S*_2_ are the nodes set of the reconstructed and actual sources respectively.

## 3 Simulation

### 3.1 Single-Source Reconstruction

In this section, the 111 single-source samples in the test set were selected for BLT reconstruction to verify the accuracy of the 1DCNN in single-source reconstruction.

**Table 2** presents the average and standard deviation of the LE and Dice in single-source reconstruction. For the Dice, 1DCNN performs 10% better than IPS, while the average LE of 1DCNN is 11.3% less than that of IPS.

**Table 2:** The average and standard deviation of the LE and Dice for the single-source reconstruction in test set.

Method	LE (mm)	Dice
IPS	0.42 ± 0.25	0.59 ± 0.18
1DCNN	0.37 ± 0.23	0.65 ± 0.18

**Figure 2:**
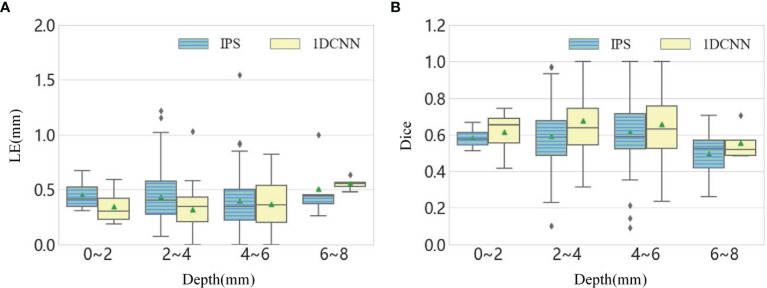
**(A)** The boxplot chart of the LE for all single-source samples, where the samples are divided into four groups according to the depth. **(B)** The corresponding boxplot chart of the Dice.

To further investigate the impacts of depth on the performance of method, we divided the 111 single-source test samples into four groups according to the depth range and analyze the LE and Dice at different depths. The statistical values are summarized into a boxplot, as shown in **Figure 2**. It can be seen that the LE slightly increases with the depth, and the average value of Dice value goes below 0.6 when the depths of source range from 6 to 8 mm. In summary, the average performance of 1DCNN is better than IPS for the samples at different depths.

**Figure 3:**
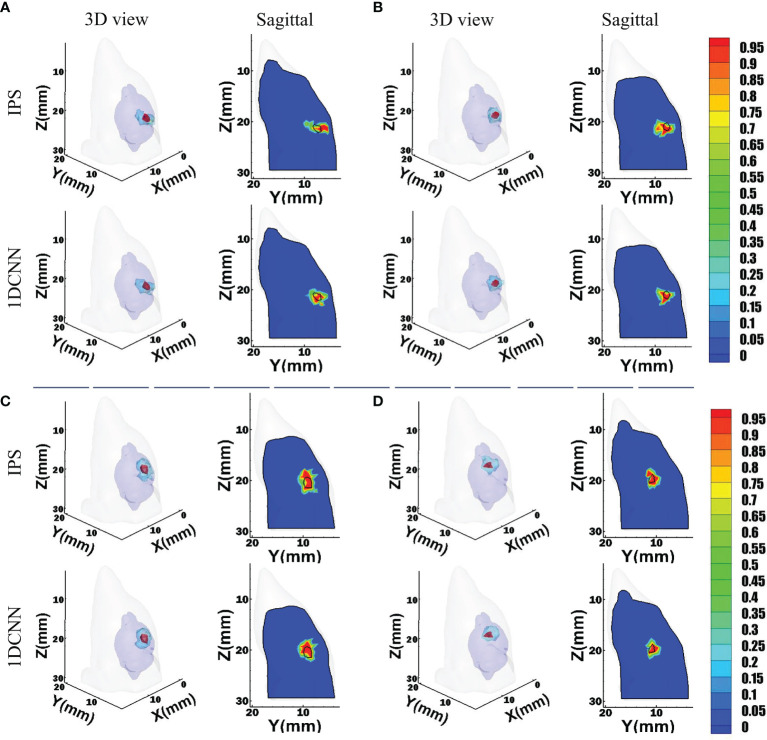
Reconstruction results of 1DCNN and IPS in the single-source case, including the 3D views and the corresponding sagittal views at the true source center: **(A)** Depth = 2.9 mm; **(B)** Depth = 3.5 mm; **(C)** Depth = 4.3 mm; **(D)** Depth = 6.7 mm.

For the convenience of intuitive assessment of the reconstruction results, we choose four groups of single-source sample to compare. These representative samples are similar in size but locate at different depths. The depths of these sources are 2.9, 3.5, 4.3, and 6.7 mm, respectively. As we can see in **Figure 3**, the 1DCNN results show better morphological similarity than the IPS method at different source depths. In contrast, more unexpected artifacts were observed in the IPS results.

### 3.2 Dual-Source Reconstruction

To evaluate the resolving power of different reconstruction methods, 824 groups of BLT reconstructions were performed on dual-source samples. **Table 3** summarizes the statistical results including the average and the standard deviation of Dice, LE for two individual sources (LE1 and LE2), and total LE for the two reconstructed sources. Compared with the IPS method, 1DCNN has obvious advantages in location accuracy and morphological similarity. The average LE for each reconstructed source is close to 0.5 mm. For the total LE, the average location error of 1DCNN is lower at 0.44 mm than that of the IPS method. The average Dice of 1DCNN increases by 38.78% relative to that of IPS.

**Table 3:** The average and standard deviation of the LE and Dice for the dual-source reconstruction in test set.

Method	LE1 (mm)	LE2 (mm)	Total LE (mm)	Dice
IPS	0.78 ± 0.38	0.69 ± 0.53	1.46 ± 0.63	0.49 ± 0.17
1DCNN	0.52 ± 0.36	0.50 ± 0.41	1.02 ± 0.45	0.68 ± 0.13

**Figure 4:**
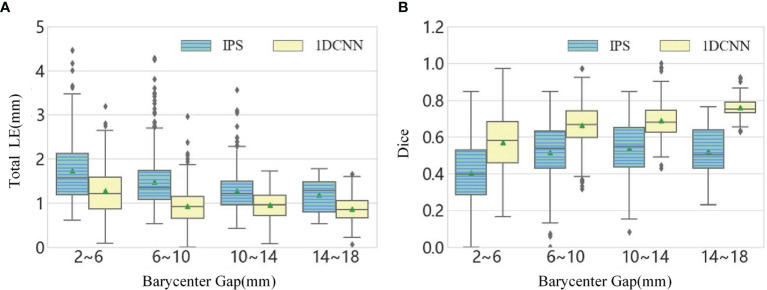
**(A)** The boxplot chart of the total LE for the dual-source samples, where the samples are divided into four classes according to the barycenter gap. **(B)** The corresponding boxplot chart of the Dice.

For dual-source reconstruction, decreasing the separation increases the difficulty of reconstruction. This can be seen in the statistical results in the boxplots of **Figure 4**, which is obtained by dividing the dual-source samples into four groups according to the separation. The total LE for dual-source samples with the separation ranging from 2 to 6 mm are the largest and the Dice index for this group is also obviously lower than the other cases. In addition, the boxplot graphs in **Figure 4** show that there was a high level of dispersion and sizable quantity of outliers within the result of IPS, especially for the total LE.

From the above statistical results, we observed that the 1DCNN method produces lower LE, which revealed that the 1DCNN method had better source location-tracing ability than the IPS method. In addition, it can be seen from the difference between the maximum and minimum values of LE in different barycenter gaps that the 1DCNN method produced more stable results.

**Figure 5:**
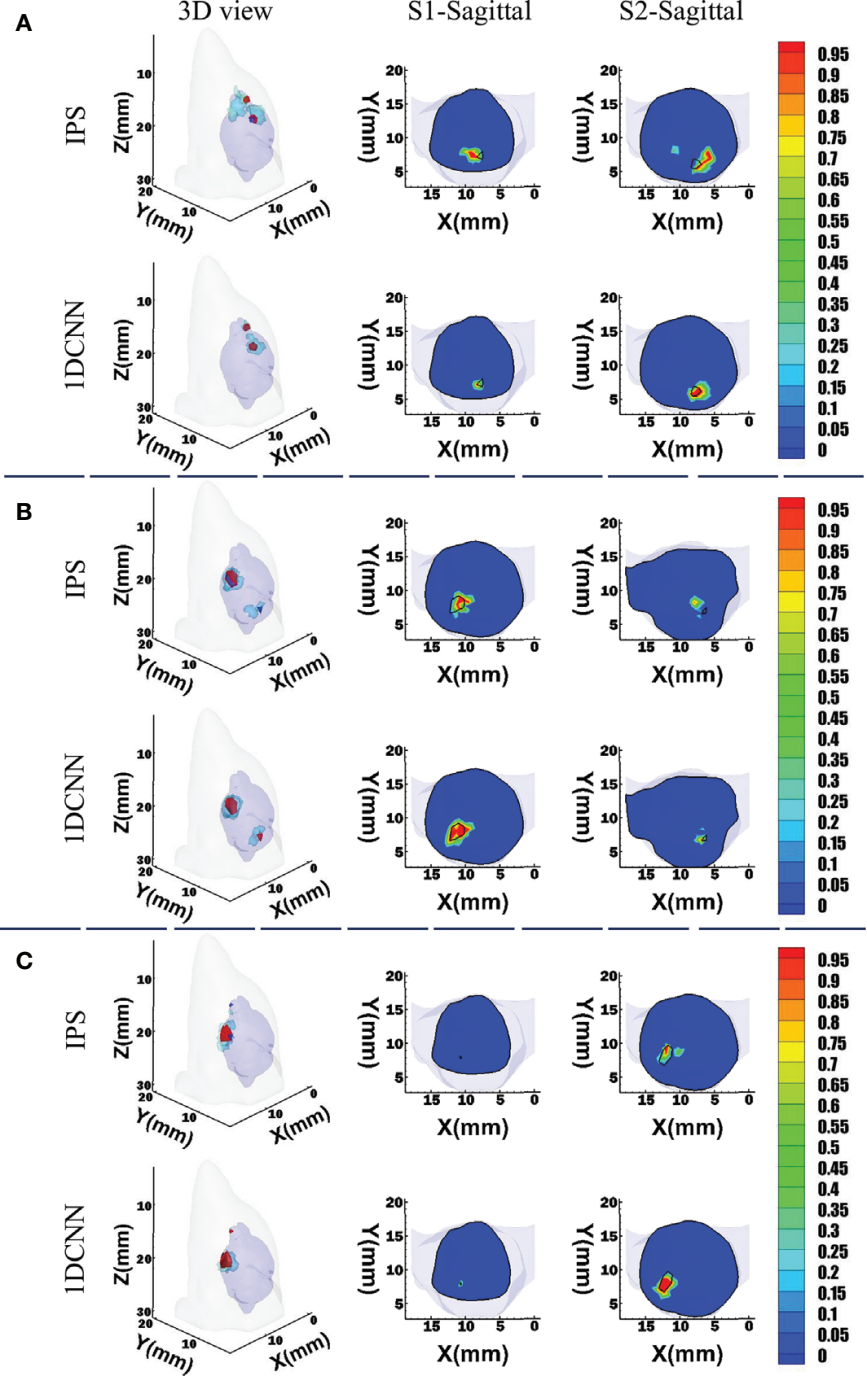
3D views and the corresponding transverse views of reconstruction results by the 1DCNN and the IPS in the dual-source cases. **(A)** Case 1; **(B)** case 2; **(C)** case 3.

For visual comparison, we randomly chose three sets of dual-source samples with different source setups. For case 1, two sources have a barycenter gap of 3.5 mm, but their sizes are similar. For cases 2 and 3, the sources have larger volume difference. **Figure 5** shows the 3D views and corresponding transverse views of the reconstruction results obtained by 1DCNN and IPS, respectively. It can be observed that both methods can reconstruct two separate sources for cases 1 and 2. However, the 1DCNN results show better morphological consistency with the true sources. In contrast, obvious position deviation and more unexpected artifacts were observed in the IPS results. For case 3, due to the huge difference in volume, IPS fails to recover the smaller source, whereas the 1DCNN successfully identifies two sources.

## 4 *In Vivo* Experiment

We further conducted BLT reconstruction in a mouse orthotopic glioma model to evaluate the practicability and the reconstruction performance of the 1DCNN method for *in vivo* animal study. A 4- to 6-week BALB/c nude mouse was prepared. Animal experiment was implemented under the guidelines approved by the Institutional Animal Care and Use Committee. To build the orthotopic glioma model, green fluorescent protein (GFP)-labeled 87MG-GFP-fLUC cells (29) were injected into the brain of the mouse. The raw data of CT was obtained by the micro-CT imaging system (UltraBright, Bolton, UK). The bioluminescent images were acquired by an electron-multiplying charge-coupled device (EMCCD) cameras (iXon888, Andor, Belfast, UK), 20 s exposure. In the process of bioluminescence image acquisition, a bandpass filter (Semrock, Rochester, NY, USA) with 670 ± 15 nm was used.T2-weighted MR images (M3TM, Aspect Imaging, Shoham, Israel) were acquired with the following parameter: TR 6,000 ms, TE 50 ms, slice thickness 0.7 mm, and slice spacing 0.2 mm.

The CT data were utilized as the structural information, and the standard mesh was registered to CT data. The cross-modal registration process of optical data and CT data were responsible for establishing the mapping relationship between the three-dimensional physical space of CT and the two-dimensional image space of BLI. We used physical markers to set six marked points in the imaging space, then we calculated the actual physical position of the camera optical center through enumeration and adjusted the mapping relationship to minimize the mapping error of the marked points. The required MRI data were used for evaluating BLT reconstruction.

**Figure 6:**
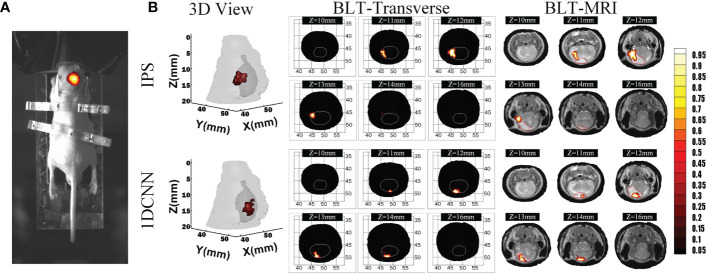
Reconstruction results in ***in vivo*** experiments. **(A)** The fusion image of the white light image and BLI. **(B)** The 3D view and several transverse section images of BLT reconstruction results and merged images of BLT and MRI data, where the MRI highlight region contour is drawn in red line.

**Figure 6A** shows the fusion images used for reconstruction, including the white light image and the bioluminescent image of the glioma-bearing mouse. The reconstructed result was merged with the corresponding MRI data by the maximum mutual information registration (30). **Figure 6B** shows the 3D view and several transverse section images of the reconstruction results and the merged images of BLT and MRI data. For visual comparison, the contour of the MRI highlight region is drawn in red lines. For quantitative analysis of *in vivo* experiments, we calculated Dice index between the different transverse section images of BLT reconstruction results and 2D MRI images of corresponding sections. Therefore, the redefinition of **Equation (9)** in the *in vivo* experiment was S1 is the BLT reconstruction result area of transverse section images and S2 is the area of the highlight region of the MRI image. **Table 4** lists the quantitative results of the calculated Dice value. The *in vivo* results revealed that the reconstructed regions given by the 1DCNN method achieved better accuracy and morphology recovery and were better overlapped with MRI highlight regions.

**Table 4:** Quantitative results of Dice for *in vivo* BLT reconstruction.

Method	z-coordinate for the selected transverse section				Mean
	*Z* = 11 mm	*Z* = 12 mm	*Z* = 13 mm	*Z* = 14 mm	
IPS	0.09	0.51	0	0	0.15
1DCNN	0.31	0.47	0.77	0.92	0.62

## 5 Discussion and Conclusion

In this paper, we propose a deep-learning method based on one-dimensional convolutional neural networks to deal with the inverse problem of BLT reconstruction. This method directly fits the nonlinear mapping relationship between the surface measurement and the internal sources to avoid iteratively solving the inverse problem based on a simplified photon transmission model. Since the local connection and weight-sharing characteristics of the convolutional neural network could reduce the number of parameters to be trained in the network model, this allows the network of 1DCNN to deal with more complex problems and achieve fast reconstruction than the IPS method.

The simulation results show that the 1DCNN method can achieve better tumor resolution, position accuracy, and morphological fitting. The dual-source results shown in **Figures 4**, **5** illustrate that not only does 1DCNN performed better than IPS in morphological fitting but it also provided better resolving ability in different source settings. The quantitative analysis in **Tables 2**, **3** shows that the proposed 1DCNN method has remarkable advantages especially in dual-source reconstruction. *In vivo* experiments have also proved the feasibility and superiority of the proposed method in tumor detection. As shown in **Figure 6**, **Table 4**, although the BLT results are not well consistent with the MRI highlight regions in part of the selected transverse views, the results of 1DCNN are generally better than that of IPS.

In conclusion, the proposed method solved the ill-posed inverse problem of BLT based on a deep-learning framework. Although the data collection stage and network training stage are time consuming, the computational burden and time cost for reconstruction are very low compared with traditional iterative inverse algorithms. However, there are still some shortcomings, such as the need for additional registration between standard meshes and the data collection scheme limiting the reconstruction accuracy. Due to the irregular shape of the digital mouse brain, the proportions of single-source samples at different depths and dual-source samples at different barycenter gaps were uneven when acquiring the dataset. For example, the dual-source samples with barycenter gap of 6–10 mm accounted for 40% of the total samples in the test set. Therefore, there are more outliers focused on those intervals, as shown in **Figure 4**. Our future work will focus on solving these problems to further improve the generalization capability and reconstruction accuracy.

## Data Availability Statement

The raw data supporting the conclusions of this article will be made available by the authors, without undue reservation.

## Ethics Statement

The animal study was reviewed and approved by the Institutional Animal Care and Use Committee.

## Author Contributions

JY and CD participated in the design of this study, and they both drafted the manuscript. JY carried out formulation of research goals, data analysis and manuscript review. CD carried out the design of computer programs, collection of data set and result visualization. XH and HG provided assistance for in vivo experiment. CD, SS and YL carried out literature search and data preprocessing. All authors contributed to the article and approved the submitted version.

## Funding

This study was funded by the National Natural Science Foundation of China (11871321, 61971350, 61901374, 61906154), Postdoctoral Innovative Talents Support Program (No. BX20180254), Natural Science Foundation of Shaanxi under Grant 2019JQ-724, and Scientific and Technological projects of Xi’an under Grant 201805060ZD11CG44.

## Conflict of Interest

The authors declare that the research was conducted in the absence of any commercial or financial relationships that could be construed as a potential conflict of interest.

## Publisher’s Note

All claims expressed in this article are solely those of the authors and do not necessarily represent those of their affiliated organizations, or those of the publisher, the editors and the reviewers. Any product that may be evaluated in this article, or claim that may be made by its manufacturer, is not guaranteed or endorsed by the publisher.
